# High Potency Allœpathy

**Published:** 1890-06

**Authors:** Charles B. Gilbert

**Affiliations:** A Physician Practicing Homœopathy—A Homœopathist


### "HIGH POTENCY ALLŒOPATHY."

CHARLES B. GILBERT, M. D.

(A Physician Practicing Homœopathy—A Homœopathist.)

*Digitalis.*—Sound in left ear as if steam was rushing through a small hole, *with a feeling as if the pharynx was enlarged;* pulse slow.

Sore throat with a feeling *as if the pharynx was wide open* and swollen; the eyes are inflamed; nose running, left side.

The enlarged feeling in pharynx is purely clinical and led to the prescription in the second case, which was promptly cured; the action was so striking that a homœopathic physician, who had opportunity to observe it, begged to know the remedy.

When one reads the statement from the compiler of a *Mat. Med. Pura,* that clinical experience is "high potency allopathy," it makes one think of the difference between riding a hobby and a hobby-horse, as given by a lunatic: "When one tires of a horse, he gets down; a man never gets down from a hobby." It would seem that there needs to be a transfer of patients between the inside and the outside.

